# Impacts of social restrictions on mental health and health behaviours of individuals with multimorbidity during Covid-19 pandemic

**DOI:** 10.1177/26335565231221609

**Published:** 2023-12-14

**Authors:** Valérie Chauvin, Resti Tito H Villarino, Paquito Bernard, Hanan Yazbek, Laurence Kern, Marie Hokayem, Lama Mattar, Gayatri Kotbagi, Melissa Rizk, Yannick Morvan, Aurélie Baillot, Ahmed Jérôme Romain

**Affiliations:** 1 5622 Université de Montréal, Montréal, QC, Canada; 2 Centre de recherche de l'Institut universitaire en santé mentale de Montréal, Montréal, QC, Canada; 3 317511 Cebu Technological University, Cebu, Philippines; 4 Université du Québec à Montréal, Montréal, QC, Canada; 5 Centre local de services communautaires, Montréal, QC, Canada; 6 27044 Université Paris-Nanterre, Nanterre, France; 7 67028 Holy Spirit University of Kaslik (USEK), Jounieh, Lebanon; 8 37607 Lebanese American University, Lebanon; 9 211516 FLAME University, Pune, India; 10 59310 Université du Québec en Outaouais, Gatineau, QC, Canada; 11 Institut du Savoir Monfort, Ottawa, ON, Canada

**Keywords:** COVID-19, social restrictions, multimorbidity, mental health, health behaviours

## Abstract

**Background:**

Social restrictions and their possible impact on lifestyle make people with multimorbidity (≥2 co-existing chronic conditions) more vulnerable to poor perceived mental health and health behaviours modifications during the COVID-19 pandemic.

**Objective:**

To understand the mental health status and health behaviour modifications among individuals with multimorbidity during different levels of COVID-19 social restrictions.

**Methods:**

Longitudinal multinational cohort study consisting of two online questionnaires with its first wave taken place while social restrictions were imposed (May 2020), and its second wave with less social restrictions in place (November 2020). Including 559 participants (wave 1) and 147 participants from wave 1 (wave 2) with an average age of 34.30±12.35 and 36.21±13.07 years old. Mostly females living in Canada, France, India and Lebanon.

**Results:**

The prevalence of multimorbidity was 27.68% (wave 1) and 35.37% (wave 2). While social restrictions were imposed, people with multimorbidity were 2 to 3 times more likely to experience psychological distress, depressive symptoms, increased stress or isolation than those without multimorbidity. Health behaviours were also modified during this period with people with multimorbidity being more likely to reduce their physical activity and increased their fruit and vegetable consumption. In wave 2, regardless of multimorbidity status, sexual desire continuously decreased while stress and psychological distress increased.

**Conclusion:**

Mental health and health behaviours modifications occurred while social restrictions were imposed and people with multimorbidity were more severely impacted than those without multimorbidity, indicating a need for a more adapted approach of care during socially restrictive periods for this population.

## Introduction

To minimize the spread of COVID-19, health regulations, such as social distancing and home confinement measures, were government-imposed in low-, middle- and high-income countries.^1–3^ In previous outbreaks (e.g., Ebola, SARS-COV1), freedom restrictions were associated with increased risks of post-traumatic stress disorder, fear, nervousness, depression, and anxiety.^4–6^ Among the stressors associated with poor mental health, the duration of confinement, fear of infection, frustration, restriction, loneliness, and difficulty providing for basic needs (e.g., food and medical supplies) were prominent.^4,7^ After social restrictions, additional stressors such as financial situation, employment, and stigma from others were found to impair mental health over time.^4,6,8,9^

During the COVID-19 pandemic, people with chronic conditions such as hypertension, diabetes and obesity were found to have higher risk of severe and fatal COVID-19.^10–12^ In this context, people with multimorbidity (defined as the occurrence of two or more chronic conditions in the same person)^13^ were more likely to experience mental health and health behaviours modifications due to Covid-19.^14^

Multimorbidity is widely prevalent (25% prevalence worldwide)^15^ and is considered as an upgrowing burden in low- and middle-income countries (LMICs).^16^ It is explained by a rapid increase in chronic diseases combined with health systems that are not designed to manage multimorbidity.^16^ Also, the prevalence of multimorbidity is found to be higher in high-income countries (HICs)^17^ hence an international analysis of the impact of social restrictions on people with multimorbidity is necessary. Multimorbidity is associated with an increased risk of unplanned hospitalization, economic uncertainty, food insecurity,^18^ and people with multimorbidity are more likely to have poor mental health (e.g., clinical depression, anxiety, reduced well-being)^19,20^ and use health-related resources (e.g., psychologists, doctors) more frequently than the general population.^19–21^ Furthermore, people with multimorbidity are more likely to have unhealthy behaviours (e.g., sedentary lifestyle, unhealthy eating, alcohol use, smoking).^21–23^ Also, due to the COVID-19 pandemic public health measures, people were more likely to negatively change their health behaviours, such as changes in eating habits caused by restriction and reduced availability of products, and a decrease in physical activity.^6,8,24,25^

As a result, social restrictions and their possible impact on lifestyle make people with multimorbidity more likely to have poor mental health and health behaviour modifications with consequences that would persist beyond the end of the restrictions.

Hence, the objectives of the present longitudinal study were:

• To analyze the mental health status of individuals with multimorbidity and to examine the relation between mental health and health behaviours among people with multimorbidity during the presence of social restrictions,
• To evaluate the evolution in health behaviours (physical activity, sleep, alcohol, tobacco, cannabis, sexual life/desires, fruit and vegetable consumption) while imposed with social restrictions between people with or without multimorbidity,
• To examine the persistence of poor perceived mental health and health behaviours modifications with lesser COVID-19 social restrictions in place among individuals with multimorbidity.

We hypothesized that:

(H1)Individuals with multimorbidity would be more likely to experience poor perceived mental health and health behaviours modifications while imposed with social restrictions during the COVID-19 pandemic,

H2)A prevalence of 15 to 20% of poor perceived mental health (psychological distress, depressive symptoms, perceived daily stress, and isolation) with a stronger prevalence among people with multimorbidity would be found,^20^

H3)Health behaviours would not be restored with less social restrictions due to their influence on people.

## Methods

### Study-specific inclusion criteria

The inclusion criteria were to be an adult (≥18 years old; identified by date of birth) with or without physical or mental chronic pathologies, who could read and understand questionnaires in French or English, and with access to an Internet connection.

### Design

Longitudinal cohort study consisting of two waves of online questionnaires through the Limesurvey platform (a statistical survey, polling, and online form creation software). The first wave occurred, on May 14^h^, 2020, and the second wave on November 2^nd^, 2020. In terms of sampling, different researchers called on their professional networks (*Société Québécoise pour la Recherche en Psychologie*, *Société Française des Professionnels en Activité Physique Adaptée*, and more) via their mailing list and respective personal networks, as well as on social networks (Facebook, Twitter).

After answering the first survey, participants were asked whether they would like to be contacted for the longitudinal aspect of this study. If yes, they were redirected to another survey unliked to their previous answers to give their contact information. Hence, all information was anonymously surveyed throughout the study.

This study was approved by the ethics committee of the University of Montreal (#2020-143) and participants gave their consent online before having access to the questionnaires.

### Definition of physical multimorbidity and multimorbidity

In the present study, participants were asked whether they had (yes/no) a diagnosis of each of the following chronic physical pathologies: asthma, hypertension, arthritis, heart disease, diabetes, cancer, dyslipidemia, back pain, a bowel disease (Crohn’s disease, ulcerative colitis, irritable bowel syndrome or bowel incontinence), a stomach or intestinal ulcer, stroke. Obesity (body mass index being ≥30 kg/m^2^) was added to the list and calculated using participants self-reported weight and height (weight (kg)/height (m)^2^). The following mental health disorders were also assessed: mood disorder (depression, bipolar disorder, mania, or dysthymia), anxiety (phobia, obsessive-compulsive disorder or panic disorder), and psychosis or schizophrenia spectrum disorder. To be considered as chronic, a condition had to be present for at least six months (or should have lasted for more than six months) and diagnosed by a healthcare professional. The selection of chronic conditions was based on data from the Canadian Community Health Survey.

Multimorbidity, in the present study, was defined as the co-occurrence of two or more of the assessed chronic physical or mental pathologies. A physical multimorbidity subgroup was also created (defined as the presence of only two or more of the assessed chronic physical conditions) (Roberts et al., 2015). Therefore, participants could be integrated in both, the multimorbidity and physical multimorbidity subgroups if they had two or more chronic physical pathologies (e.g., diabetes and asthma). Yet, participants would only be integrated into the multimorbidity category if they had one physical and one chronic mental condition (e.g., asthma and anxiety).

### Social restrictions

As this study ran internationally, the social restriction measures in place varied depending on the country of residency. By social restrictions, we included any social and sanitary measures that were in place due to the COVID-19 pandemic and the time of this study. In this section we describe the social restrictions in place in the four countries from which more than one participant from our study resides.

During Wave 1, in Canada, social restrictions varied by provinces and territories and included social distancing and mask wearing. Large gatherings were forbidden (ex., ≥ 10 people) and most nonessential stores were closed. In France, social restrictions included the obligation to wear a mask in transport and social distancing. Restaurants and most places of large gathering were closed and travels beyond 100 km were prohibited. In India, social restriction included confinement to partial confinement depending on the zone of residency within the country. The entire country needing to follow social distancing rules as well as wearing a mask with large gathering strictly prohibited. In Lebanon, social restrictions included complete confinement of the population.

During Wave 2, in Canada, social restriction varied by provinces and territories and included social distancing and mask wearing, while most stores were open, with large gathering still forbidden but less restricted than during wave 1. Restaurants were mostly open with takeouts and delivery available, some provinces had a system with variations in the level of social restrictions depending on the location of residency within the country. In France, open establishments had a limit of occupancy allowing social distancing of 4m^2^ per person with a gathering limitation of 6 people yet allowing professional gathering and transport. In India, there were in their Unlock 6.0 phase with a partial reopening of schools and reopening of public transport and touristic activities, compared to wave 1 where the confinements were in place. In Lebanon, a nationwide curfew from 9pm to 5am was in effect for the population with individuals being limited to their town of residency as many towns and villages were under confinement, yet the population itself was allowed to exit their residency compared to wave 1.

### Mental health markers

The mental health markers evaluated were perceived psychological distress, depressive symptoms, perceived daily stress and perceived isolation.

Psychological distress was assessed using the K6 scale.^26^ This scale has scores ranging from 0 to 24, and higher scores represent a higher presence of psychological distress. In our study, a standard score ≥ 13 was considered as a high level of psychological distress.

Depressive symptoms were assessed using the Center for Epidemiological Studies Depression Scale (CES-D). The CES-D is valid and reliable for discriminating between people with and without depressive symptoms.^27–30^ Its score ranges from 0 to 60, where the cut-off scores to be considered as having depressive symptoms were ≥ 17 for males and ≥ 23 for female participants.^31^

Perceived daily stress was assessed using a single item from the Canadian Community Health Survey where participants were asked: “*Thinking about the amount of stress in your life, would you say that most of your days are*…”. Five options were offered being: not at all stressful, not very stressful, a little stressful, quite a bit stressful and extremely stressful ranging from 1 to 5. Participants with scores ≥ 4 were considered as being stressed.

Perceived isolation was assessed using the perceived isolation scale.^32^ Participants were asked how often they felt like they were running out of company, they felt left out or isolated with the options using a 5-point Likert scale. Participants with scores ≥ 4 were considered as feeling isolated.

### Socio-demographic factors

The socio-demographic factors were age, sex, and gender. Regarding the participants’ residency, the access to an exterior or interior garden or balcony was assessed. Also, given the surveys were launched internationally, the country where they live was assessed. Countries of residency were then classified using the World Bank current classification by income.^33^ For example, Canada and France were classified as high-income countries (HIC) and India and Lebanon as low-middle income countries (LMIC). Participants’ marital status (single, married, spouse/couple or divorced/separated) and level of employment (unemployed/part-time/retired, full time or students) were also assessed.

### Health behaviours

The different health behaviours assessed were physical activity, alcohol, tobacco, cannabis, nutrition, sleep and sexual life/desires. In the first wave (May 2020), participants were asked to assess all the previously mentioned health behaviours with regards to the previous year, then to assess their present behaviour during the presence of the social restrictions. In the second wave, participants were asked to assess their behaviour at the time of the survey (November 2020). All health behaviours except physical activity and sexual life were assessed with items from the Canadian Community Health Survey.

Physical activity was assessed with two items recommended to evaluate usual physical activity in population studies^34^ using both frequency and duration. Participants were asked to include sports, fitness, or leisure time physical activities which lasted at least 10 minutes continuously (e.g., walking, biking, running).

Alcohol consumption was assessed in terms of frequency during the last month and over the last year. This item classified the frequency of alcoholic beverages consumption with the options being: less than once a month, once a month, 2 to 3 times a month, once a week, 2 to 3 times a week, 4 to 6 times a week, and every day with scores ranging from 1 to 7.

Cannabis was assessed with two items regarding its use over the year and month prior to the present study. Those items were: *“Have you used cannabis (yes/no) in the past 12 months?”,* with “cannabis” referring to marijuana, hashish, hash oil or any other product of the cannabis plant. The same questions were applied to the last month. Then, the frequency of this behaviour was assessed with the options being: less than once a month, 1 to 3 times a month, once a week, more than once a week and every day or almost every day, with scores ranging from 1 to 5.

Tobacco consumption was assessed with two items. Participants were asked if they were active smokers and, if yes, to provide the number of cigarettes per day.

Nutrition was assessed with 4 items on fruits and vegetables consumption. Those items being: *“In the past month, excluding the juices, have you eaten any vegetables?”* and *“How many times a week have you eaten vegetables?”* both for fruits and vegetables and for the different period evaluated.

Sleep was assessed with four items assessing the duration of sleep (hours/night), the restorative capacity of sleep with *“How often is your sleep refreshing?”*, problems falling asleep with *“How often do you have problems falling asleep or staying asleep?"* and daytime sleepiness with *“How often do you have difficulty staying awake when you want to?”* with the options: never, rarely, sometimes, most of the time and all the time ranging from 1 to 5.

Sexual life was assessed with two items focused on sexual desire, described as a feeling that includes the desire to have sexual activity, being receptive to a partner's sexual advances, and having thoughts or fantasies about the sexual act .^35^ Nevertheless, desire was not necessarily related to the presence of a partner. Participants reported their frequency (almost never or never, rarely, sometimes, most of the time, almost always or always ranging from 1 to 5) and their average level (very weak or nonexistent, low, medium, high, and very high ranging from 1 to 5).

### Statistical analyses

Descriptive statistics were used to categorize participants. In a first step, to analyze the impact of the social restrictions on mental health (perceived psychological distress, depressive symptoms, perceived daily stress and perceived isolation), logistic regression models were run. These models had the mental health markers as dependent variables and the presence of multimorbidity/physical multimorbidity as independent variable, and were adjusted for age, sex, countries income ranking, housing, and employment.

In a second step, to analyze whether potential associations observed at step 1 were modified by the health behaviours (physical activity, alcohol, tobacco, cannabis, fruits and vegetable, sleep, and sexual desire) during the presence social restrictions, the logistic regression models were also adjusted on these variables. These models had the same variables as in the first step, with the health behaviours added as additional covariates.

Then, to analyze the evolution in health behaviours regarding the multimorbidity/ physical multimorbidity status across time, a repeated-measures linear mixed model using an unstructured covariance structure^36^ was run and was adjusted for age, sex, countries income ranking, housing and employment. When significant, post-hoc analysis was performed.

Finally, to analyze the persistence of poor perceived mental health and health behaviours modifications, descriptive analyses and linear mixed models with the same parameterization as in the previous steps were run while including the data from the second wave. These analyses were run only with participants who answered the questionnaires from both waves.

All results were analyzed according to multimorbidity and physical multimorbidity, respectively.

### Missing data management

Statistical analyzes were run on the imputed version of the dataset to decrease statistical bias due to missingness. All statistical models were performed using both, the original and imputed dataset and given no difference emerged between the original and imputed dataset, only results from the imputed dataset were reported.

Level of data missingness was 0.96% (ranging between 0%-2.78% for each variable) in wave 1 and 4.59% (ranging between 0%-2.78% for each variable 0-20.40%) in wave 2.

Statistical analyses were run on RStudio (1.4.1106) with the packages “ISLR”^37^, “emmeans”^38^, “nlme”^39^, “VIM”^40^, “mice”^41^ and “haven”.^42^

### Transparency

All R syntaxes used to realize the present study are available at https://osf.io/z2ve5/.

## Results

### Wave 1

#### Sample characteristics

The first wave included 559 participants (444 women, 80.44%), with a mean age of 34.30±12.35 years old. Most had access to a garden or an interior courtyard (n = 267, 48.63%) or a balcony (n = 214, 38.98%). Most were living in a HIC (n = 358, 67.17%), were married, common-law partners or in a relationship (n = 291, 53.01%), and were either full-time workers (n = 263, 48.52%) or students (n = 184, 33.95%) (Table 1).

**Table 1.** Descriptive characteristics of the population.

	Wave 1				Wave 2*			
	N	%	M	Std. Dev	N	%	M	Std. Dev
Sex								
Male	108	19.57			25	17.00		
Female	444	80.43			119	80.95		
N/A	7	-			3	-		
Age			34.30	12.35			36.21	13.07
Housing								
Housing with garden or interior courtyard	267	48.63			71	48.30		
Accommodation with balcony but without garden	214	38.98			59	40.14		
Accommodation without exterior access	68	12.39			17	11.56		
N/A	10	-			0	-		
Marital status								
Single	238	43.35			55	37.41		
Married / common-law partner / couple	291	53.01			84	57.14		
Divorced / separated	20	3.64			7	4.76		
N/A	10	-			1	-		
Employment								
Unemployment / Part-time / Retired	95	17.53			36	24.49		
Full time	263	48.52			67	46.57		
Student	184	33.95			41	27.89		
N/A	17	-			3	-		
Countries income ranking								
Lower-middle income countries	175	32.83			25	17.00		
Higher income countries	358	67.17			115	78.23		
N/A	26	-			7	-		

* Participants from wave 2 answered the surveys for both waves.

### Rate of multimorbidity and physical multimorbidity

The prevalence of multimorbidity within the first wave was 27.68% (n = 150) with 23.86% (n = 42) in LMICs and 29.08% (n = 105) in HICs. The prevalence of physical multimorbidity was 20.85% (n = 113), with 14.20% (n = 25) in LMICs and 23.82% (n = 86) in HICs (See Supplementary file A for the prevalence in wave 2). It is to be noted that the number of participants from the LMICs and HICs does not equate the total number of participants as some did not report their country of residency (n = 3). The prevalence of each individual disease is in Supplementary file B.

### Rate of the mental health outcomes during social restrictions

Within the first wave, the prevalence of psychological distress was 19.25% (n = 97), 35.70% (n = 176) for depressive symptoms, 31.80% (n = 159) for stress and 19.16% (n = 96) for feelings of isolation.

### Association between mental health markers and multimorbidity or physical multimorbidity during social restrictions

#### Psychological distress

Participants with multimorbidity were twice more likely to report psychological distress (OR 2.48, 95%CI [1.57-3.92], p < 0.001). Similar results were found for physical multimorbidity (OR 1.88, 95%CI [1.12-3.12], p = 0.01) (Table 2).

**Table 2.** Association between mental health according to multimorbidity status.

Multimorbidity					Physical multimorbidity				
	OR	95% IC		P value		OR	95% IC		P value
Psychological distress					Psychological distress				
Age	0.99	0.97	1.01	0.84	Age	0.99	0.97	1.02	0.93
Sex	1.94	1.08	3.70	**0.03**	Sex	1.95	1.09	3.70	**0.03**
Countries’ income rank	0.56	0.35	0.90	**0.01**	Countries’ income rank	0.56	0.35	0.89	**0.01**
Housing	1.59	1.16	2.19	**0.003**	Housing	1.59	1.16	2.17	**0.003**
Employment	0.98	0.69	1.41	0.94	Employment	0.99	0.70	1.41	0.97
Multimorbidity	2.48	1.57	3.92	**<0.001**	Physical multimorbidity	1.88	1.12	3.12	**0.01**
Depressive symptoms					Depressive symptoms				
Age	0.96	0.94	0.98	**<0.001**	Age	0.96	0.94	0.96	**<0.01**
Sex	1.28	0.80	2.06	0.29	Sex	1.27	0.81	2.03	0.29
Countries’ income rank	0.89	0.60	1.33	0.58	Countries ‘income rank	0.88	0.60	1.31	0.55
Housing	1.16	0.89	1.51	0.26	Housing	1.15	0.89	1.50	0.26
Employment	0.93	0.69	1.26	0.66	Employment	0.94	0.71	1.26	0.72
Multimorbidity	3.09	2.07	4.64	**<0.001**	Physical multimorbidity	1.84	1.18	2.85	**0.006**
Stress					Stress				
Age	0.99	0.98	1.01	0.76	Age	0.99	0.98	1.01	0.81
Sex	1.38	0.87	2.23	0.17	Sex	1.40	0.88	2.26	0.15
Countries’ income rank	0.74	0.49	1.10	0.14	Countries’ income rank	0.72	0.48	1.08	0.11
Housing	0.92	0.70	1.21	0.57	Housing	0.93	0.71	1.21	0.61
Employment	0.82	0.61	1.11	0.20	Employment	0.83	0.62	1.11	0.22
Multimorbidity	2.33	1.58	3.44	**<0.001**	Physical multimorbidity	2.10	1.36	3.23	**<0.001**
Isolation					Isolation				
Age	0.97	0.95	0.99	**0.03**	Age	0.97	0.95	1.00	**0.05**
Sex	1.96	1.09	3.77	**0.03**	Sex	1.96	1.09	3.75	**0.03**
Countries’ income rank	1.18	0.74	1.90	0.47	Countries’ income rank	1.18	0.74	1.90	0.47
Housing	1.29	0.95	1.75	0.09	Housing	1.29	0.95	1.75	0.09
Employment	0.97	0.69	1.37	0.88	Employment	0.97	0.70	1.37	0.89
Multimorbidity	1.92	1.21	3.01	**0.004**	Physical multimorbidity	1.26	0.74	2.10	0.37

When adjusted on health behaviours, associations were slightly reduced (OR 2.36, 95%CI [1.44-3.85], p < 0.001) for multimorbidity but remained similar for physical multimorbidity. Physical activity and cannabis consumption were significantly associated (Table 3).

**Table 3.** Association between mental health according to multimorbidity status and health behaviours.

Multimorbidity					Physical multimorbidity				
	OR	95% IC		P Value		OR	95% IC		P Value
Psychological distress					Psychological distress				
Age	1.00	0.98	1.02	0.66	Age	1.00	0.98	1.03	0.58
Sex	1.61	0.85	3.18	0.15	Sex	1.61	0.86	3.18	0.14
Countries’ income rank	0.61	0.35	1.05	0.07	Countries’ income rank	0.61	0.35	1.06	**0.07**
Housing	1.81	1.28	2.57	**<0.001**	Housing	1.81	1.29	2.57	**<0.001**
Employment	1.00	0.69	1.47	0.97	Employment	1.61	0.70	1.49	0.91
Physical activity*	0.40	0.24	0.67	**<0.001**	Physical activity*	0.39	0.23	0.65	**<0.001**
Alcohol	0.96	0.84	1.09	0.56	Alcohol	0.95	0.83	1.09	0.52
Tobacco	1.02	0.97	1.06	0.31	Tobacco	1.02	0.97	1.07	0.29
Cannabis	1.65	1.36	1.99	**<0.001**	Cannabis	1.65	1.37	2.00	**<0.001**
Nutrition	0.98	0.95	1.00	0.28	Nutrition	1.98	0.95	1.00	0.24
Sleep problems	1.23	0.91	1.66	0.16	Sleep problems	1.24	0.92	1.67	0.15
Sexual life	0.85	0.67	1.07	0.17	Sexual life	0.84	0.67	1.06	0.16
Multimorbidity	2.36	1.44	3.85	**<0.001**	Physical multimorbidity	1.88	1.08	3.25	**0.02**
Depressive symptoms					Depressive symptoms				
Age	0.96	0.94	0.98	**<0.001**	Age	0.96	0.94	0.98	**0.002**
Sex	0.96	0.57	1.62	0.88	Sex	0.95	0.57	1.59	0.85
Countries’ income rank	0.94	0.59	1.50	0.81	Countries’ income rank	0.96	0.60	1.52	0.86
Housing	1.19	0.89	1.59	0.22	Housing	1.18	0.89	1.57	0.23
Employment	0.96	0.70	1.33	0.83	Employment	0.97	0.71	1.33	0.89
Physical activity*	0.62	0.41	0.93	**0.02**	Physical activity*	0.60	0.40	0.88	**0.01**
Alcohol	0.93	0.83	1.03	0.18	Alcohol	0.91	0.82	1.02	0.11
Tobacco	1.04	1.00	1.08	**0.04**	Tobacco	1.04	1.00	1.09	**0.03**
Cannabis	1.38	1.17	1.63	**<0.001**	Cannabis	1.38	1.17	1.63	**<0.001**
Nutrition	0.99	0.98	1.00	0.38	Nutrition	0.99	0.97	1.00	0.38
Sleep problems	1.82	1.41	2.37	**<0.001**	Sleep problems	1.81	1.41	2.35	**<0.001**
Sexual life	1.04	0.86	1.27	0.62	Sexual life	1.04	0.86	1.26	0.65
Multimorbidity	2.96	1.93	4.56	**<0.001**	Physical multimorbidity	1.75	1.09	2.81	**0.01**
Stress					Stress				
Age	0.99	0.97	1.01	0.60	Age	0.99	0.97	1.01	0.67
Sex	1.09	0.66	1.80	0.72	Sex	1.10	0.67	1.81	0.70
Countries’ income rank	0.71	0.45	1.12	0.15	Countries’ income rank	0.71	0.45	1.11	0.13
Housing	0.86	0.64	1.14	0.30	Housing	0.87	0.65	1.15	0.33
Employment	0.76	0.55	1.03	0.08	Employment	0.77	0.56	1.05	0.09
Physical activity*	0.60	0.41	0.89	**0.01**	Physical activity*	0.60	0.41	0.88	**<0.001**
Alcohol	0.96	0.86	1.06	0.47	Alcohol	0.95	0.86	1.06	0.41
Tobacco	1.00	0.96	1.04	0.72	Tobacco	1.00	0.96	1.04	0.71
Cannabis	0.99	0.84	1.17	0.95	Cannabis	1.00	0.85	1.18	0.93
Nutrition	0.98	0.97	1.00	0.13	Nutrition	0.98	0.96	0.99	0.11
Sleep problems	1.45	1.14	1.85	**0.002**	Sleep problems	1.46	1.15	1.86	**0.001**
Sexual life	0.81	0.67	0.98	**0.02**	Sexual life	0.81	0.68	0.98	**0.03**
Multimorbidity	2.25	1.50	3.37	**<0.001**	Physical multimorbidity	2.04	1.30	3.21	**0.001**
Isolation					Isolation				
Age	0.97	0.95	1.00	**0.05**	Age	0.97	0.95	1.00	0.08
Sex	1.76	0.93	3.53	0.09	Sex	1.72	0.91	3.44	0.10
Countries’ income rank	1.32	0.77	2.29	0.30	Countries’ income rank	1.35	0.79	2.34	0.26
Housing	1.29	0.93	1.78	0.12	Housing	1.28	0.93	1.77	0.12
Employment	1.06	0.74	1.53	0.74	Employment	1.05	0.74	1.52	0.75
Physical activity*	0.69	0.43	1.10	0.13	Physical activity*	0.67	0.42	1.07	0.10
Alcohol	0.88	0.77	1.00	0.06	Alcohol	0.87	0.77	0.99	**0.04**
Tobacco	1.06	1.01	1.10	**0.007**	Tobacco	1.06	1.01	1.11	**0.004**
Cannabis	1.19	0.99	1.43	**0.05**	Cannabis	1.19	0.99	1.42	**0.05**
Nutrition	0.99	0.97	1.00	0.57	Nutrition	0.99	0.97	1.00	0.58
Sleep problems	1.61	1.20	2.16	**0.001**	Sleep problems	1.61	1.21	2.17	**0.001**
Sexual life	1.34	1.06	1.69	**0.01**	Sexual life	1.33	1.06	1.68	**0.01**
Multimorbidity	1.72	1.06	2.76	**0.02**	Physical multimorbidity	1.10	0.62	1.90	0.72

* Physical activity was dichotomised (0/1) according to meeting or not participants the recommendations of ≥150min/week.^43^

#### Depressive symptoms

Participants with multimorbidity were three times more likely to report depressive symptoms (OR 3.09, 95%CI [2.07-4.64], p < 0.001). When looking at physical multimorbidity, they were almost twice more likely (OR 1.84, 95%CI [1.18-2.85], p = 0.006) (Table 2).

When adjusted on health behaviours, the associations were slightly reduced for both subgroups (multimorbidity: OR 2.96, 95% CI [1.93-4.56], p < 0.001; physical multimorbidity: OR 1.75, 95% CI [1.09-2.81], p = 0.01) with physical activity, sleep, cannabis, and tobacco consumption being significant for both groups.

#### Stress

Participants with multimorbidity and physical multimorbidity were twice more likely to feel stressed daily (Multimorbidity: OR 2.33, 95%CI [1.58-3.44], p < 0.001; Physical multimorbidity: OR 2.10, 95%CI [1.36-3.23], p < 0.001) (Table 2).

When adjusted on health behaviours, the odds ratio were slightly reduced for both subgroups but remained similar (Multimorbidity: OR 2.25, 95%CI [1.50-3.37], p < 0.001; Physical multimorbidity: OR 2.04, 95%CI [1.09-2.81], p = 0.001) with physical activity, sleep and sexual life being significant for both groups.

#### Feelings of isolation

Participants with multimorbidity were almost twice more likely to feel isolated (OR 1.92, 95%CI [1.21-3.01], p = 0.004) while no association was found for people with physical multimorbidity (Table 2).

When adjusted on health behaviours, the odds ratios were slightly reduced (OR 1.72, 95% CI [1.06-2.76], p = 0.02) for multimorbidity and there was no association of physical multimorbidity with cannabis consumption, sleep and sexual life being significant for both groups and with alcohol and tobacco being significant only for physical multimorbidity (Table 3).

### Evolution of health behaviours before versus during social restrictions

**Figure 1.**
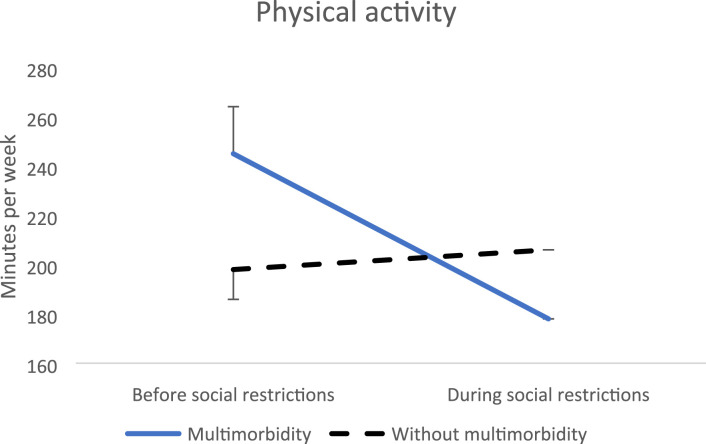
Evolution of physical activity before versus during social restrictions.

Regarding physical activity, a reduction of weekly physical activity duration was observed during the presence of social restrictions (-74,41, SE = 27,65, p = 0.007) (Figure 1). The post-hoc indicated that people with multimorbidity decreased their physical activity level during wave 1. Regarding physical multimorbidity, no significant results were found.

**Figure 2.**
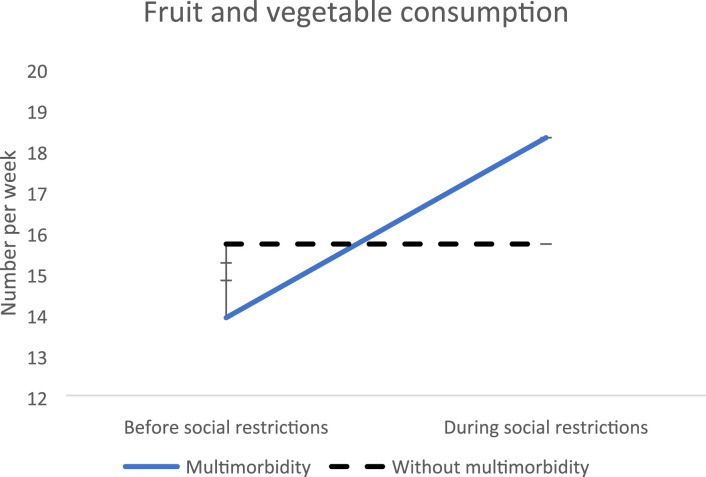
Evolution of fruit and vegetable consumption before versus during social restrictions.

Regarding nutrition, an increase in weekly fruit and vegetable consumption was found (4.38, SE = 1.8, p = 0.01) (Figure 2). In people with physical multimorbidity, the increase was more pronounced (6.12, SE = 1.99, p = 0.002), with an increase from 14 to 20 intake of fruit or vegetable per week. However, the post-hoc for multimorbidity and physical multimorbidity were not significant.

**Figure 3.**
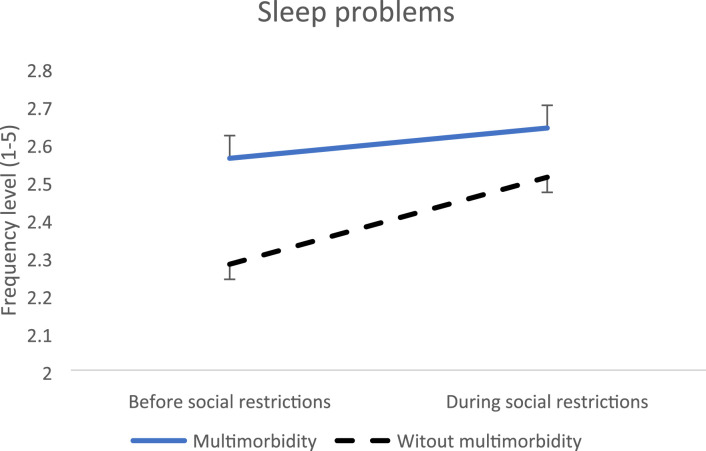
Evolution of sleep problems before versus during social restrictions.

Regarding sleep problems (less sleep capacity, more problems falling asleep and more daytime sleepiness), an increase was found regardless multimorbidity status, yet being less pronounced in people with multimorbidity (-0.15, SE = 0.06, p = 0.01) (Figure 3). However, the post-hoc indicated that people with multimorbidity had higher levels of sleep problems before social restrictions (-0.28, SE = 0.06, p < 0.01). When analyzing for physical multimorbidity, results were not significant.

Regarding the other health behaviours, no differences were found either for people with multimorbidity or physical multimorbidity: alcohol (p = 0.39; p = 0.72), tobacco (p = 0.23; p = 0.75), cannabis (p = 0.16; p = 0.53) and sexual desires (p = 0.15; p = 0.21) (Supplementary file C). For the evolution of all the health behaviours see the supplementary files D and E.

### Wave 2

#### Sample characteristics

The second wave included 147 participants (women: n = 119, 80.95%) among those polled in wave 1 (abandon rate = 79.60%) with a mean age of 36.21±13.07 years old, who had access to a garden or an interior courtyard (n = 71, 48.30%) or a balcony (n = 59, 40.14%), and living in a HIC (n = 115, 78.23%). Most were married, common-law partners or in a relationship (n = 84, 57.14%) and either full-time workers (n = 67, 46.57%) or students (n = 41, 27.89%) (Table 1).

#### Prevalence of multimorbidity and physical multimorbidity

The prevalence of multimorbidity was 35.37% (n = 52) and 28.57% (n = 42) for physical multimorbidity.

#### Evolution of the prevalence of the mental health markers

When analyzing data from participants who answered questionnaires from both waves 1 and 2, the prevalence of psychological distress increased from 17.36% (n = 25) to 18.36% (n = 27), stress increased from 27.59% (n = 40) to 39.46% (n = 58). However, depressive symptoms decreased from 40.69% (n = 59) to 33.33% (n = 49), and isolation decreased from 21.53% (n = 31) to 19.73% (n = 29).

#### Evolution of health behaviours in wave 2

**Figure 4.**
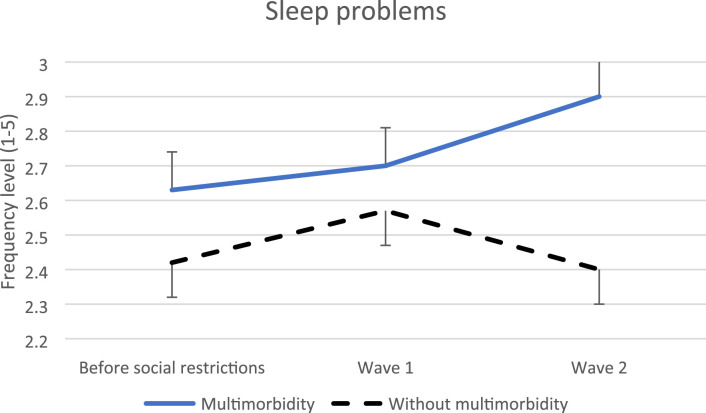
Evolution of sleep problems before social restrictions versus during wave 1 and 2.

Regarding sleep problems, an increase was found during wave 2 (0.29, SE = 0.14, p = 0.05) (Figure 4) and the post-hoc indicated higher level of sleep problems for people with multimorbidity in wave 2. Regarding physical multimorbidity, a wave 2 increase (0.31, SE = 0.15, p = 0.04) was also observed. The post-hoc indicated that sleep problems were systematically higher for people with physical multimorbidity.

**Figure 5.**
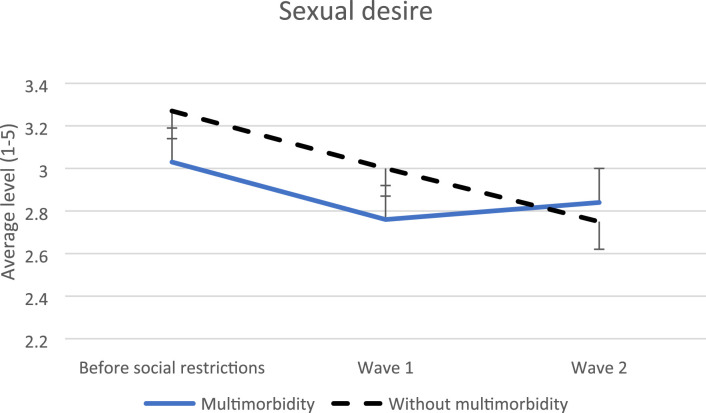
Evolution of sexual desires before social restrictions versus during wave 1 and 2.

Regarding sexual desire, a decrease in the level was noted for both people with and without multimorbidity, being more pronounced among people without multimorbidity (0.33, SE = 0.16, p = 0.04) (Figure 5). In people with physical multimorbidity, no modification was found (p = 0.14).

Regarding the other health behaviours, no differences were found either for multimorbidity or physical multimorbidity for physical activity (p = 0.82; p = 0.17), alcohol (p = 0.21; p = 0.17), tobacco (p = 0.95; p = 0.84), cannabis (p = 0.72; p = 0.77) and fruit and vegetable consumption (p = 0.48; p = 0.21) (Supplementary file F). For the evolution of all the health behaviours see the supplementary files G and H.

## Discussion

The objectives of the present study were to analyze the impact of COVID-19 social restrictions on mental health and health behaviours of individuals with multimorbidity and physical multimorbidity and to verify whether health behaviours attenuated poor perceived mental health. Also, our study aimed to compare the evolution in health behaviours while imposed with social restrictions according to participants’ multimorbidity status and to assess the persistence of behaviour modifications longitudinally at a time with lesser social restriction measures.

For the first wave, the prevalence of multimorbidity and physical multimorbidity was almost 28 and 21%, respectively which is in accordance with the worldwide prevalence data and therefore validates our sample. The lower percentage for physical multimorbidity in our study compared to the international data of 25% can be explained by our young population as it is known that the prevalence of multimorbidity increases with age.^44^ As expected in the literature, the prevalence of multimorbidity was higher in HICs (28%) than in LMICs (23%).^17^ The same observation goes for physical multimorbidity with 21% in HICs and 14% in LMICs. Yet, it is to be noted that the prevalence of multimorbidity is high and concerning in all countries surveyed. Regarding poor perceived mental health, we found that 19% of our sample had psychological distress, 35% depressive symptoms, 31% stress and 19% felt isolated, which were higher than hypothesized. So, as in previous studies,^4,45^ our study clearly demonstrates a concerning level of poor perceived mental health at a time of social restrictions and shows the importance to consider the mental health of individuals throughout pandemic periods.

Besides, our study showed that people with multimorbidity were two to three times more likely to experience psychological distress, depressive symptoms, stress, and feelings of isolation while imposed with social restrictions which validates our hypothesis. Nevertheless, these results were expected as a meta-analysis^46^ underlined that people with non-communicable chronic diseases were more likely to report poor perceived mental health, including psychological distress and depression, compared their counterparts without chronic diseases. Also, similar results were found for people with physical multimorbidity, yet tendencies were slightly lower for psychological distress, depressive symptoms, and stress, while showing no association with feelings of isolation. These differences are potentially due to the co-occurrence between physical and mental health pathologies given our study included chronic mental health conditions in the multimorbidity group. However, this assumption was not explored in the present study given only 37 participants had both physical and mental conditions making it too small of a sample to explore. Here we would recommend studies on complex multimorbidity (co-occurrence of physical and mental health conditions). Interestingly, a study including 550 people with mental disorders (57% psychotic disorders, 33% mood disorders) showed no impact of the social restrictions on depressive symptoms which was explained by the habitual high rates of social isolation in this population and the general slowdown in social activity.^45^

Interestingly, tendencies of people with multimorbidity to experience psychological distress, depressive symptoms, feelings of isolation and stress while imposed with social restrictions were slightly reduced when our models were adjusted on health behaviours. Health behaviours, particularly physical activity, had a protective effect on these psychological markers. This observation is supported given it is well known that a healthy lifestyle (e.g., physical activity, nutrition, sleep) can have a protective effect on mental health.^47–49^ Interestingly, results remained similar for physical multimorbidity, except for psychological distress and feelings of isolation which could be explained by the fact our study did not survey behaviours aiming to reduce feelings of isolation (e.g., communications through phone calls or videoconferences) and that certain health behaviours (e.g., nutrition, physical activity) have social aspects which were disrupted by the public health measures in place.

Regarding the evolution of mental health markers in wave 2, depressive symptoms decreased by 6% and feelings of isolation by less than 1%, while the prevalence of stress increased by 13%, and psychological distress by 1%. The decrease in both depressive symptoms and feelings of isolation is not surprising as previous findings showed an association between these two markers among young adults out of the pandemic.^50^ Also, the second wave took place at a time allowing an increase in social interactions which goes along with the reduction in feelings of isolation in many countries. However, other sociosanitary measures were still in place (e.g., social distancing). The increase in stress and psychological distress could be explained by factors such as the financial situation and employment ^4,6,8,9^ which remained precarious due to the uncertainty of the sociosanitary situation at the time, even more knowing that stress is a factor allowing adaptation to situations that present challenges.^51^

Regarding health behaviours, a reduction of physical activity was found while social restrictions were imposed, and people with multimorbidity decreased their weekly physical activity levels by 67 minutes per week, while people without multimorbidity maintained their physical activity levels with a negligible 8-minute increase. Interestingly, when analysing participants who participate in both waves, both people with and without multimorbidity decreased their physical activity during the first wave, followed by a significant increase in wave 2 and surpassed their baseline values before the social restrictions. Here, people with multimorbidity decreased their physical activity more than people without multimorbidity during wave 1 while reporting a greater increase in wave 2. Although self-reported physical activity can be overestimated, our result shows a clear negative impact of the social restrictions on physical activity levels of all individuals, as in a previous study,^52^ though being more pronounced for people with multimorbidity. Moreover, all tendencies were maintained for physical multimorbidity.

Regarding fruits and vegetable consumption, as in a previous study,^53^ an increase was found when considering multimorbidity and physical multimorbidity versus people without multiple chronic pathologies. However, another study found that people with obesity, one of the conditions included in multimorbidity, were prone to lower the frequency of their fruit and vegetable consumption while increasing their food consumption and snacking during confinement.^54^ In the present study, we did not survey other nutrition behaviours (e.g., snaking), therefore we cannot confirm rather this increase in fruit and vegetable consumption was not associated with an increase in general food consumption.

Additionally, an increase in perceived sleep problems was found in participants, regardless their multimorbidity status. This increase was lower for people with multimorbidity knowing that they reported as much sleep problem than people without multimorbidity while imposed with social restrictions yet reported higher levels of habitual pre-restriction sleep problems. These findings are consistent with studies showing that sleep patterns, quality of sleep and insomnia levels were negatively impacted during the COVID-19 outbreak.^55,56^

Meanwhile, there were no significant differences in behaviour adaptation between people with and without multimorbidity nor physical multimorbidity regarding alcohol, tobacco, and cannabis consumption as well as sexual desires while imposed with social restrictions. However, a study found that people with higher levels of depression and anxiety reported less sexual activity during confinement.^57^ Indeed, it is also worthy to mention that overall sense of boredom worsened by a significant decrease or lack of social interactions, loss of daily structure, no true feeling of fulfillment after a hard-working day, and decreased conviviality which was explained as the primary causes for the increased intake of various substances (alcohol and cigarettes) which was not the case in our study.^58^

Regarding the health behaviours in wave 2, their evolution from the pre-COVID period to presence of social restrictions showed an increase in sleep problems during wave 2 among people with multimorbidity. In fact, people with multimorbidity constantly increased their level of sleep problems throughout the study, being at its highest in wave 2, while people without multimorbidity had their sleep problem-level peak during wave 1 yet came back to baseline value in wave 2. A longitudinal study underlined that sleep problems decreased after the reduction of public health measures^59^ and went in the same direction as our finding concerning people without multimorbidity.^60,61^

Regarding sexual desire in wave 2, people with and without multimorbidity had a lower sexual desire level compared to the pre-social restrictions’ period. Interestingly, previous studies reported a decrease in sexual desire among women^62,63^ (which represents 80% of our population) during social restrictions, and no study reported the persistence of this decrease longitudinally. Hence, our study suggests that sexual desire continuously decreased after the imposition of social restrictions with no regard to the presence of chronic diseases and would need to be better analyzed in further longitudinal studies. When looking at physical multimorbidity, there was no significant difference found.

Meanwhile, there were no significant differences in behaviour adaptation between people with and without multimorbidity nor physical multimorbidity in wave 2 regarding physical activity, alcohol, tobacco, cannabis and fruit and vegetable consumption. People with multimorbidity or physical multimorbidity and those without multimorbidity all decreased their physical activity levels in wave 1 then increase drastically in wave 2. They all increased their alcohol consumption in wave 1 then decreased in wave 2. Regarding tobacco and cannabis, they slightly increased their number of cigarettes per day in wave 2 and slightly decreased their cannabis consumption. Finally, they all increased their fruit and vegetable consumption in wave 1 than decreased it in wave 2. Though interesting, it is important to note that the present data illustrate the health behaviours following the presence of social restrictions which were surveyed at a time of hope for many and was done without knowing that additional confinement periods and social restrictions measures would be imposed. Longitudinal studies reporting the impact of repeated social restrictive periods on health behaviours would be needed to ensure better division and access to behaviour-related health services (e.g., kinesiology, nutrition) throughout pandemic periods.

In light of our findings, people with multimorbidity would benefit from special monitoring during and following social restrictions or pandemic periods in order to limit their mental health deterioration. Indeed, our study highlights the needs of new services related to social restrictions or similar circumstances such as climatic events. A recent study showed that climatic events such as air pollution, extreme temperatures and natural disasters could have negative effects on physical activity which were also found to be more important in adults with chronic diseases.^64^ Consequently, the results suggest orienting the available professional resources and guiding future interventions (for example, offering the right resource - kinesiologist, nutritionist, psychologist, etc.) to the right person throughout crisis.

A strength of the present study is the analysis of two types of multimorbidity; multimorbidity defined by physical chronic diseases and concurrent mental health disorders and multimorbidity defined solely by physical chronic diseases. Another strength is the self-reported aspect of the assessed mental health markers. Lastly, the present study reports data from participants mainly from Canada, France, Lebanon and India, while also including a small number of participants from Uganda, Australia, Singapore, Israel, United Kingdom, Switzerland, Tunisia and the Netherlands. It allows an international overview of the impact of multimorbidity and physical multimorbidity during and following social restrictions.

Regardless, several limitations should be acknowledged. Firstly, since our study took place through online surveys, health behaviours were self-reported, probably leading to bias in estimation. However, in a will to survey people internationally and due to the sociosanitary measures in places during this study, only self-reported measures were feasible and necessary. Secondly, there is a limit regarding the sample’s representativeness in relation to the general population caused by the recruitment channels used, the high abandon rate between the first and second wave and the high presence of females in our sample. Thirdly, the consumption of psychiatric medication was not surveyed in this study while the consumption of antidepressants, anxiolytics, and sedative-hypnotic drugs increased, especially in women, during the COVID-19 pandemic.^65^ Fourthly, all cannabis-related findings could be modified by the Canadian legislation of cannabis as Canadian present a large proportion of the population studied and that cannabis is not legal in the other countries surveyed.^66^ Finally, the level to which individuals adhered to the social restrictions were not surveyed in this study and could have varied between countries and between individuals which could have had an impact on our findings.

### Conclusion

Ultimately, our results further reinforce that social restrictions negatively altered mental health and health behaviour in people with multimorbidity and that people with multimorbidity are more vulnerable those negative consequences beyond the implementation of social restrictions. Consequently, there is a need to create and adapt interventions of care for this population.

## Supplemental Material

Supplemental Material - Impacts of social restrictions on mental health and health behaviours of individuals with multimorbidity during Covid-19 pandemic Click here for additional data file. Supplemental Material for Impacts of social restrictions on mental health and health behaviours of individuals with multimorbidity during Covid-19 pandemic by Chauvin Valérie, Villarino Resti Tito H, Bernard Paquito, Yazbek Hanan, Kern Laurence, Hokayem Marie, Mattar Lama, Kotbagi Gayatri, Rizk Melissa, Morvan Yannick, Baillot Aurélie and Romain Ahmed Jérôme in Journal of Multimorbidity and Comorbidity
