# Supplementary material for: Impacts of social restrictions on mental health and health behaviours of individuals with multimorbidity during Covid-19 pandemic
Source: J Multimorb Comorb. 2023 Dec 14;13:26335565231221609. doi: 10.1177/26335565231221609 (PMC10725145; doi:10.1177/26335565231221609)
Supplement: Supplemental Material - Impacts of social restrictions on mental health and health behaviours of individuals with multimorbidity during Covid-19 pandemic [file sj-pdf-1-cob-10.1177_26335565231221609.pdf]

SUPPLEMENTARY FILES

Supplementary file A: Prevalence of multimorbidity and physical multimorbidity.

	Wave 1		Wave 2	
	N	%	N	%
**Multimorbidity**	150	27.68	52	35.37
HICs	105	29.08	42	36.52
LMICs	42	23.86	7	28.00
N/A	17	-	412	-
**Physical multimorbidity**	113	20.85	42	28.57
HICs	86	23.82	34	29,50
LMICs	25	14.20	6	24.00
N/A	17	-	412	-

Supplementary file B: Prevalence of physical and mental chronic pathologies.

Chronic pathology	Wave 1		Wave 2	
	N	%	N	%
**Asthma**				
Yes	54	10.02	18	12.41
No	485	89.98	127	87.59
N/A	20	-	2	-
**Hypertension**				
Yes	36	6.65	12	8.22
No	505	93.35	134	91.78
N/A	18	-	1	-
**Arthritis**				
Yes	27	5.02	11	7.59
No	511	94.98	134	92.41
N/A	21	-	2	-
**Diabetes**				
Yes	9	1.66	4	2.74
No	533	98.34	142	97.26
N/A	17	-	1	-
**Stroke**				
Yes	1	0.18	1	0.69
No	540	99.82	144	99.31
N/A	18	-	2	-
**Heart disease**				
Yes	27	5.02	7	4.86
No	511	94.98	137	95.14

N/A	21	-	3	-
**Chronic back pain**				
Yes	146	27.29	44	30.34
No	389	72.71	101	69.66
N/A	24	-	2	-
**Dyslipidemia**				
Yes	16	2.99	4	2.78
No	519	97.01	140	97.22
N/A	24	-	3	-
**Cancer**				
Yes	11	2.03	6	4.11
No	531	97.97	140	95.89
N/A	17	-	1	-
**Bowel disease**				
Yes	45	8.40	15	10.34
No	491	91.60	130	89.66
N/A	23	-	2	-
**Stomach or intestinal ulcer**				
Yes	10	1.86	3	2.05
No	529	98.14	143	97.95
N/A	20	-	1	-
**Obesity**				
Yes	86	15.81	26	17.81
No	458	84.19	120	82.19
N/A	15	-	1	-
**Mood disorder**				
Yes	53	10.08	20	13.99
No	473	89.92	123	86.01
N/A	33	-	4	-
**Anxiety disorder**				
Yes	73	13.85	21	14.79
No	454	86.15	121	85.21
N/A	32	-	5	-
**Psychosis**				
Yes	2	0.37	1	0.69
No	537	99.63	143	99.31
N/A	20	-	3	-

Supplementary file C: Comparison in behaviour adaptation before versus during social restrictions between people with multimorbidity and physical multimorbidity.

Multimorbidity				Physical multimorbidity			
	Value	Std. Error	P value		Value	Std. Error	P value
**Health behaviour**				**Health behaviour**			
Physical activity	-74.42	27.65	**0.007**	Physical activity	-54.60	30.74	0.07
Alcohol	0.10	0.12	0.39	Alcohol	0.05	0.13	0.72
Tobacco	0.24	0.20	0.23	Tobacco	0.07	0.23	0.75
Cannabis	-0.08	0.05	0.16	Cannabis	-0.04	0.06	0.53
Fruits/Vegetables	4.38	1.80	**0.01**	Fruits/Vegetables	6.12	1.99	0.002
Sleep	-0.16	0.06	**0.01**	Sleep	-0.09	0.07	0.18
Sexual desire	-0.10	0.07	0.15	Sexual desire	-0.10	0.08	0.21

Supplementary file D: Evolution of health behaviours before versus during social restrictions for people with and without multimorbidity.

		**Multimorbidity**		**No multimorbidity**	
**Health behaviour**	**Social restrictions**	**Mean**	**SE**	**Mean**	**SE**
**Physical activity** *Minutes/week*	Before	245	19.1	198	12.7
	Wave 1	178	19.1	206	12.7
**Alcohol** *Frequency levels (1-7)*	Before	2.77	0.15	3.07	0.10
	Wave 1	2.84	0.15	3.04	0.10
**Tobacco** *Number of cigarettes/days*	Before	2.35	0.40	1.04	0.28
	Wave 1	2.89	0.40	1.33	0.27
**Cannabis** *Frequency levels (1-5)*	Before	1.51	0.10	1.55	0.07
	Wave 1	1.59	0.10	1.71	0.07
**Fruits/Vegetables** *Frequency of consumption/week*	Before	13.9	1.34	15.7	0.89
	Wave 1	18.3	1.34	15.7	0.89
**Sleep problems** *Frequency levels (1-5)*	Before	2.56	0.06	2.28	0.04
	Wave 1	2.64	0.06	2.51	0.04
**Sexual desire** *Average level (1-5)*	Before	3.36	0.07	3.38	0.05
	Wave 1	3.06	0.07	3.18	0.05

Supplementary file E: Evolution of health behaviours before versus during social restrictions for people with and without physical multimorbidity.

		Physical multimorbidity		No physical multimorbidity	
**Health behaviour**	**Social restrictions**	**Mean**	**SE**	**Mean**	**SE**
**Physical activity**	Before	238	22.1	204	12.2
Minutes/week	Wave 1	181	22.1	202	12.2
**Alcohol**	Before	2.77	0.17	3.05	0.10
Frequency levels (1-7)	Wave 1	2.80	0.17	3.03	0.09
**Tobacco**	Before	2.32	0.46	1.17	0.27
Number of cigarettes/days	Wave 1	2.74	0.46	1.51	0.27
**Cannabis**	Before	1.40	0.12	1.58	0.06
Frequency levels (1-5)	Wave 1	1.50	0.12	1.72	0.07
**Fruits/Vegetables**	Before	14.2	1.54	15.5	0.86
Frequency of consumption/week	Wave 1	20.3	1.54	15.5	0.86
**Sleep problems**	Before	2.53	0.07	2.32	0.04
Frequency levels (1-5)	Wave 1	2.64	0.07	2.52	0.04
**Sexual desire**	Before	3.39	0.09	3.37	0.05
Average level (1-5)	Wave 1	3.09	0.09	3.16	0.05

Supplementary file F: Comparison in behaviour adaptation before social restrictions versus during wave 1 and wave 2 between people with multimorbidity and physical multimorbidity.

**Multimorbidity**				**Physical multimorbidity**			
	Value	Std. Error	P value		Value	Std. Error	P value
**Health behaviour**				**Health behaviour**			
Physical activity	38.06	169.07	0.82	Physical activity	63	178.86	0.72
Alcohol	0.31	0.24	0.21	Alcohol	0.36	0.26	0.17
Tobacco	0.06	1.04	0.95	Tobacco	0.20	1.10	0.84
Cannabis	-0.02	0.06	0.72	Cannabis	-0.01	0.66	0.77
Fruits/Vegetables	-1.03	1.47	0.48	Fruits/Vegetables	-1.94	1.55	0.21
Sleep problems	0.29	0.14	**0.05**	Sleep problems	0.31	0.15	**0.04**
Sexual desire	0.33	0.16	**0.04**	Sexual desire	0.25	0.17	0.14

Supplementary file G: Health behaviours before social restrictions versus during wave 1 and wave 2 for people with and without multimorbidity.

		Multimorbidity		No multimorbidity	
**Health behaviour**	**Social restrictions**	**Mean**	**SE**	**Mean**	**SE**
**Physical activity** *Minutes/week*	Before	220	108.2	151	92.6
	Wave 1	185	108.2	148	92.6
	Wave 2	405	108.2	299	92.6
**Alcohol** *Frequency levels (1-7)*	Before	2.95	0.25	3.46	0.22
	Wave 1	3.28	0.25	3.70	0.22
	Wave 2	2.08	0.25	2.28	0.22
**Tobacco** *Number of cigarettes/days*	Before	1.47	0.82	0.51	0.72
	Wave 1	1.27	0.92	1.03	0.72
	Wave 2	1.93	0.82	0.91	0.72
**Cannabis** *Frequency levels (1-5)*	Before	1.12	0.08	1.10	0.08
	Wave 1	1.24	0.08	1.13	0.08
	Wave 2	1.03	0.08	1.03	0.08
**Fruits/Vegetables** *Frequency of consumption/week*	Before	17.2	4.08	21.7	3.51
	Wave 1	28.4	4.08	22.6	3.51
	Wave 2	15.3	4.08	20.8	3.51
**Sleep problems** *Frequency levels (1-5)*	Before	2.63	0.11	2.42	0.10
	Wave 1	2.70	0.11	2.57	0.10
	Wave 2	2.90	0.11	2.40	0.10
**Sexual desire** *Average level (1-5)*	Before	3.03	0.16	3.27	0.13
	Wave 1	2.76	0.16	3.00	0.13
	Wave 2	2.84	0.16	2.75	0.13

Supplementary file H: Health behaviours before social restrictions versus during wave 1 and wave 2 for people with and without physical multimorbidity.

		Physical multimorbidity		No physical multimorbidity	
**Health behaviour**	**Social restrictions**	**Mean**	**SE**	**Mean**	**SE**
**Physical activity** *Minutes/week*	Before	220	118.4	158	89.1
	Wave 1	198	118.4	146	89.1
	Wave 2	426	118.4	301	89.1
**Alcohol** *Frequency levels (1-7)*	Before	2.95	0.27	3.40	0.21
	Wave 1	3.19	0.27	3.69	0.21
	Wave 2	2.14	0.27	2.23	0.21
**Tobacco** *Number of cigarettes/days*	Before	1.68	0.90	0.50	0.70
	Wave 1	1.32	0.90	1.01	0.70
	Wave 2	2.25	0.90	0.86	0.70
**Cannabis** *Frequency levels (1-5)*	Before	1.14	0.09	1.10	0.07
	Wave 1	1.21	0.09	1.16	0.07
	Wave 2	1.04	0.09	1.03	0.07

**Fruits/Vegetables** *Frequency of consumption/week*	Before	17.3	4.46	21.1	3.37
	Wave 1	31.1	4.46	21.9	3.37
	Wave 2	14.6	4.46	20.4	3.37
**Sleep problems** *Frequency levels (1-5)*	Before	2.71	0.12	2.40	0.09
	Wave 1	2.82	0.12	2.53	0.09
	Wave 2	3.02	0.12	2.39	0.09
**Sexual desire** *Average level (1-5)*	Before	2.97	0.16	3.27	0.13
	Wave 1	2.71	0.16	3.01	0.13
	Wave 2	2.76	0.16	2.80	0.13
